# Impact of treatment intensity on infectious complications in patients with acute myeloid leukemia

**DOI:** 10.1007/s00432-022-03995-2

**Published:** 2022-05-18

**Authors:** Romy Tober, Ulf Schnetzke, Maximilian Fleischmann, Olaposi Yomade, Karin Schrenk, Jakob Hammersen, Anita Glaser, Christian Thiede, Andreas Hochhaus, Sebastian Scholl

**Affiliations:** 1 Klinik Für Innere Medizin II, Abteilung Hämatologie Und Internistische Onkologie, Universitätsklinikum Jena, Am Klinikum 1, 07747 Jena, Germany; 2 Institut Für Humangenetik, Universitätsklinikum Jena, Am Klinikum 1, 07747 Jena, Germany; 3 Universitätsklinikum Carl Gustav Carus an der Technischen Universität Dresden, Dresden, Germany

**Keywords:** AML, Infections, Epigenetic therapy, Induction chemotherapy, IFD, Pneumonia

## Abstract

**Background:**

Infectious complications reflect a major challenge in the treatment of patients with acute myeloid leukemia (AML). Both induction chemotherapy and epigenetic treatment with hypomethylating agents (HMA) are associated with severe infections, while neutropenia represents a common risk factor. Here, 220 consecutive and newly diagnosed AML patients were analyzed with respect to infectious complications dependent on treatment intensity and antifungal prophylaxis applied to these patients.

**Patients and methods:**

We retrospectively analyzed 220 patients with newly diagnosed AML at a tertiary care hospital between August 2016 and December 2020. The median age of AML patients undergoing induction chemotherapy (*n* = 102) was 61 years (25–76 years). Patients receiving palliative AML treatment (*n* = 118) had a median age of 75 years (53–91 years). We assessed the occurrence of infectious complication including the classification of pulmonary invasive fungal disease (IFD) according to the EORTC/MSG criteria at diagnosis and until day 100 after initiation of AML treatment. Furthermore, admission to intensive care unit (ICU) and subsequent outcome was analyzed for both groups of AML patients, respectively.

**Results:**

AML patients subsequently allocated to palliative AML treatment have a significantly higher risk of pneumonia at diagnosis compared to patients undergoing induction chemotherapy (37.3% vs. 13.7%, *P* < 0.001) including a higher probability of atypical pneumonia (22.0% vs. 10.8%, *P* = 0.026). Furthermore, urinary tract infections are more frequent in the palliative subgroup at the time of AML diagnosis (5.1% vs. 0%, *P* = 0.021). Surprisingly, the incidence of pulmonary IFD is significantly lower after initiation of palliative AML treatment compared to the occurrence after induction chemotherapy (8.4% vs. 33.3%, *P* < 0.001) despite only few patients of the palliative treatment group received *Aspergillus spp.*-directed antifungal prophylaxis. The overall risk for infectious complications at AML diagnosis is significantly higher for palliative AML patients at diagnosis while patients undergoing induction chemotherapy have a significantly higher risk of infections after initiation of AML treatment. In addition, there is a strong correlation between the occurrence of pneumonia including atypical pneumonia and pulmonary IFD and the ECOG performance status at diagnosis in the palliative AML patient group. Analysis of intensive care unit (ICU) treatment (e.g. in case of sepsis or pneumonia) for both subgroups reveals a positive outcome in 10 of 15 patients (66.7%) with palliative AML treatment and in 15 of 18 patients (83.3%) receiving induction chemotherapy. Importantly, the presence of infections and the ECOG performance status at diagnosis significantly correlate with the overall survival (OS) of palliative AML patients (315 days w/o infection vs. 69 days with infection, *P* 0.0049 and 353 days for ECOG < 1 vs. 50 days for ECOG > 2, *P* < 0.001, respectively) in this intent-to-treat analysis.

**Conclusion:**

The risk and the pattern of infectious complications at diagnosis and after initiation of AML therapy depends on age, ECOG performance status and subsequent treatment intensity. A comprehensive diagnostic work-up for identification of pulmonary IFD is indispensable for effective treatment of pneumonia in AML patients. The presence of infectious complications at diagnosis contributes to an inferior outcome in elderly AML patients.

**Supplementary Information:**

The online version contains supplementary material available at 10.1007/s00432-022-03995-2.

## Introduction

Infectious complications play a pivotal role in patients with acute myeloid leukemia (AML) and contribute to morbidity and mortality of AML patients significantly. There is a broad spectrum of infectious complications ranging from blood stream infections (BSI) potentially associated with severe sepsis to fungal infections regularly presenting as pneumonia. Treatment-related mortality (TRM) following induction chemotherapy with cytarabine in combination with daunorubicin occurs in about 5% of AML patients, while the major cause of fatal outcome is associated with BSI (Fernandez et al. 2009; Classen et al. 2021). It remains difficult to estimate the pre-treatment risk of TRM which makes clinical evaluation especially in elderly AML patients even more important (Walter et al. 2011). The rate of distinct complications (e.g., admission to intensive care unit, mechanic ventilation or dialysis) in AML patients undergoing intensive chemotherapy has been extensively reviewed in a large cohort of AML patients considering both baseline characteristics and dynamic parameters during AML treatment (Atallah et al. 2007).

BSI can regularly be detected in AML patients presenting with neutropenic fever and severe sepsis is associated with higher mortality in neutropenic patients (Conn et al. 2017; Malagola et al. 2008). BSI differs between patients with hematological diseases compared to those patients with solid tumors (Marin et al. 2014; Legrand et al. 2012). Multi-resistance of Gram-negative bacteria has been demonstrated to be a key factor for increased mortality and prolonged hospitalization (Sostarich et al. 2008). Colonization with carbapenem-resistant Enterobacteriaceae results in a significant increase of early death in AML patients undergoing induction chemotherapy (Ballo et al. 2019).

Pneumonia can be diagnosed in more than 25% of AML patients during induction chemotherapy (Garcia et al. 2013; Specchia et al. 2003). While pulmonary infiltrates should be detected by low-dose computed tomography, recommended diagnostic work-up does also includes bronchoalveolar lavage (BAL) to specify the pathogens of opportunistic infections (Gerritsen et al. 2017; Maschmeyer et al. 2015).

Invasive fungal diseases (IFD) is classified according to the EORTC/MSG recommendations and have a high impact on morbidity and prognosis in AML patients undergoing intensive chemotherapy (Pauw et al. 2008; Livio et al. 2010). The risk of pulmonary IFD in AML patients is associated with severity and duration of neutropenia while such important clinical factors as age, renal or hepatic dysfunction affect the risk of death in AML patients who develop IFD (Hammond et al. 2010; Neofytos et al. 2013). In a large prospective multi-center registry, multivariate analysis revealed the impact of performance status, body weight and chronic obstructive pulmonary disease on the occurrence of IFD in AML patients (Caira et al. 2015). Genetic factors of innate immune system might contribute to the susceptibility of infectious complications including pulmonary IFD in AML patients undergoing induction chemotherapy (Schnetzke et al. 2015; Fischer et al. 2016). Antifungal prophylaxis with posaconazole has been established in AML induction chemotherapy and in patients with acute graft-versus-host disease following allogeneic stem cell transplantation (ASCT) improving overall survival (Cornely et al. 2007; Ullmann et al. 2007).

While several factors contributing to the risk of severe infectious complications in AML patients have been identified, the severity of neutropenia (i.e., absolute neutrophils count below 500/µL) at diagnosis is significantly associated with duration of neutropenia, bloodstream infections and subsequently early death following induction chemotherapy of AML (Buckley et al. 2014). Furthermore, unresolved neutropenia represents a risk factor to develop IFD in neutropenic patients while early lymphopenia results in a higher risk of febrile neutropenia (Kontoyiannis et al. 2018; Ray-Coquard et al. 2003). In AML patients undergoing intensive induction chemotherapy, long-term neutropenia is regularly associated with treatment-related toxicity (e.g., mucositis) contributing to a pronounced risk for bloodstream infections (Yao et al. 2017).

Conversely, elderly AML patients receiving palliative AML therapy based on epigenetic treatment approaches show less therapy-associated toxicity but they regularly present with IFD due to low absolute neutrophil counts (e.g. due to an antecedent myelodysplastic syndrome, MDS) (Kim et al. 2020). In addition, functional impairment of neutrophils in those patients with previous MDS contributes to the enhanced susceptibility towards severe infections in elderly AML patients (Fianchi et al. 2012; Schuster et al. 2018).

Infection-related mortality can not only be observed in AML patients undergoing intensive induction chemotherapy but is also demonstrated in elderly AML patients receiving epigenetic treatment. So far, only few data exist on infectious complications of AML patients undergoing palliative treatment (Dombret et al. 2015; Kantarjian et al. 2012). Recently, Jalbut and colleagues analyzed early infectious complications in AML patients either undergoing induction chemotherapy or treatment with hypomethylating agents (Jalbut et al. 2018).

Based on a retrospective single-center analysis of 220 consecutive newly diagnosed AML patients, we sought to investigate the impact of potential risk factors (e.g., treatment regimen, antifungal prophylaxis) on the frequency and severity of infectious complications.

## Patients and methods

### Patient cohort and informed consent

A total of 220 consecutive AML patients were identified at the Department of Hematology and Oncology, University Hospital Jena, Germany. Diagnosis and start of induction chemotherapy, epigenetic treatment or cytoreductive chemotherapy were between August 2016 and December 2020. The patient cohort considered all patients with newly diagnosed AML during this period including those AML patients treated with cytoreductive chemotherapy combined with best supportive care. Patients´ characteristics are indicated in Table 1 and summarized in the CONSORT diagrams (Figure S1 and S2).

**Table 1:** Patients’ characteristics at diagnosis of AML for patients receiving palliative treatment or AML induction chemotherapy

	Palliative treatment	Induction chemotherapy	*P*
	*N* = 118	*N* = 102	
Median age at diagnosis, years (range)	75 (53–91)	61 (25–76)	< 0.001
Female patients, *n* (%)	53 (44.9)	57 (55.9)	n.s
Performance status, n (%)			< 0.001
ECOG < 1	58 (49.2)	75 (73.5)	
ECOG > 2	60 (51.8)	27 (26.5)	
Co-morbidity index, *n* (%)			< 0.001
mCCI < 1	62 (52.5)	80 (78.4)	
mCCI > 2	56 (47.5)	22 (21.6)	
WBC, per µL (range)	7.6 (0.5–320)	8,7 (0.6–331)	n.s
Hb, mmol/L (range)	5.4 (2.4–8.4)	5,6 (2.8–9.2)	
PLT, per µL (range)	54 (0–297)	62.5 (6–864)	
ANC, per µL (range)	1600 (0–62,300)	1270 (60–55,000)	
Percentage of blasts, % (range)			n.s
Peripheral blood	12 (0–89)	20 (0–96)	
Bone marrow	53 (20–95)	60 (20–98)	
FAB subtype, n (%)			n.s
M1/M2	81 (68.6)	66 (64.7)	
M4/M5	35 (29.7)	34 (33.3)	
M6	2 (1.7)	2 (1.9)	
History of AML			n.s
de novo AML	60 (50.8)	58 (56.9)	
Antecedent MDS	29 (24.6)	24 (23.5)	
Antecedent CMML or MPN	23 (19.5)	14 (13.7)	
Therapy-related AML	6 (5.1)	6 (5.9)	
Previous therapy of MDS, CMML or MPN, *n* (%)			n.s
HMA	16 (13.6)	18 (17.6)	
Ruxolitinib and/ or hydroxyurea	8 (6.8)	4 (3.9)	
Lenalidomide	–	1 (1)	
ASCT	–	2 (2)	
ELN 2017 risk group, *n* (%)			0.025
Favorable	16 (13.6)	26 (25.5)	
Intermediate	41 (34.7)	30 (29.4)	
Unfavorable	39 (33.1)	44 (43.1)	
Not available	22 (18.6)	2 (2)	

*AML* acute myeloid leukemia, *ANC* absolute neutrophil count, *ASCT* allogeneic stem cell transplantation, *CCI* Charlson comorbidity index, *CMML* chronic myelomonocytic leukemia, *FAB* French-American-British classification, *ECOG* Eastern Cooperative oncology Groups score, *ELN* European LeukemiaNET classification, *Hb* hemoglobin level, *HMA* hypomethylating agent, *MDS* myelodysplastic syndrome, *MPN* myeloproliferative neoplasia, *PLT* platelet count, *WBC* white blood count

Performance status and co-morbidity index have been documented according to the criteria published by the Eastern Cooperative Oncology Group (ECOG) and by application of a modified version of the Charlson co-morbidity index, respectively (Kelly and Shahrokni 2016; Ostgård et al. 2010).

All patients were included in the SAL registry (Study Alliance Leukemia). Patients gave their written informed consent for data acquisition after pseudonymization and analysis in the SAL registry. The participation in the AML registry of the SAL study group and the retrospective analysis presented here have been approved separately by the Ethical review committee of the University Hospital Jena and was performed in accordance with the provisions of the Declaration of Helsinki.

### Patient treatment

Induction chemotherapy was applied according to the 7 + 3 regimen or according to the OSHO protocol. In detail, patients underwent induction chemotherapy with cytarabine (100 mg/sqm as continuous 24-h infusion days 1–7) and daunorubicin (60 mg/sqm day 3–5) after inclusion in the DaunoDouble trial of the SAL study group (Röllig et al. 2020). According to the OSHO protocol, AML patients up to the age of 60 years were treated with idarubicin (12 mg/sqm, day 1–3) and intermediate-dosed cytarabine (1 g/sqm bid, day 1, 3, 5 and 7), while patients over 60 years received mitoxantrone (10 mg/sqm, day 1–3) and intermediate-dosed cytarabine (1 g/sqm bid, days 1, 3, 5 and 7) as induction chemotherapy (Büchner et al. 2012; Kahl et al. 2016).

HMA treatment consisted of either subcutaneously (s.c.) applied 5-Azacytidine (75 mg/sqm day 1–7 of a 28-day cycle) or decitabine (20 mg/sqm day 1–5 of a 28-day cycle) administered intravenously while low-dosed cytarabine (LODAC) was given s.c. (e.g. 40 mg day 1–7 every 4 weeks).

Best supportive care (BSC) regimens included infectious prophylaxis, treatment of infections and transfusion of blood products if indicated.

### Antimicrobial prophylaxis

Standard regimens consisted of acyclovir (400 mg BID) and cotrimoxazole (960 mg BID twice a week or 960 mg three times a week). Antibacterial prophylaxis with ciprofloxacin (500 mg BID) and antifungal prophylaxis with either fluconazole (200 mg BID) or posaconazole (300 mg each day after a loading dose) was initiated in case of ANC below 1000/µL at diagnosis or under treatment, respectively.

### Screening, diagnostic setup and empiric treatment

Screening for relevant nosocomial pathogens (e.g. 3MRGN or VRE) was performed regularly. In case of fever, diagnostic work-up included physical examination, X-ray or mostly computed tomography of the chest and analysis of blood cultures, screening for urinary tract infections (UTI) and clinical evaluation of central venous catheters.

Standard therapy in patients with neutropenic fever consisted of immediate initiation of intravenous antibiotic treatment with piperacillin/tazobactam. In those patients previously screened positive of 3MRGN or in case of hypotonic RR values despite rapid application of intravenous fluids with the necessity of vasopressors, antibiotic treatment was escalated to meropenem.

### Definition of neutropenia, neutropenic fever and analysis of toxicity

Neutropenia was defined as absolute neutrophil count (ANC) below 1000/µL and ANC was further categorized accordingly when ANC was below 500/µL or below 100/µL. Neutropenic fever was characterized by a body temperature of higher than 38.3 °C or of at least 38.0 °C sustaining for 1 h in the presence of absolute neutrophil counts less than 1000/µL.

### Definition of infectious events

By means of clinical and microbiological investigation, sites of infection were classified, e.g. as blood stream infection, catheter-related infection or gastrointestinal (GI) tract infection. The following infectious complications analyzed in this study are described in more detail.

#### Fever of unknown origin (FUO)

Fever of unknown origin (FUO) was defined as fever according to the above defined criteria in the absence of an identifiable focus of infection provided that during the current fever episode no other site of infection could be defined later.

#### Urinary tract infection (UTI)

Urinary tract infections (UTI) were documented as “proven UTI” in case of at least 10^5^ colony-forming units (cfu) per ml in microbiological analyses or as “probable UTI” in symptomatic patients and at least 10^4^ cfu/ ml but not exceeding 10^5^ cfu/ ml.

#### Blood stream infections and sepsis criteria

Blood cultures were routinely collected in case of fever and positive samples were monitored for antimicrobial susceptibility according to local laboratory protocols and standards. Positivity concerning skin pathogens was defined when peripheral and central venous line (CVL) blood cultures were tested positive. An infection of CVL in case of positive CVL and peripheral blood cultures (e.g. for Coagulase-negative Staphylococci, CoNS) was documented when time-to-positivity (TTP) was at least 2 h shorter for CVL blood culture compared to peripheral blood culture unless peripheral samples were tested negative. The clinical event systemic inflammatory response syndrome (SIRS) and sepsis were defined according to the guidelines published by the German Sepsis Society in 2020 (Brunkhorst et al. 2020).

#### Classification of pneumonia and fungal infections

Pneumonia was defined as a new infiltrate on chest radiograph (X-ray and/ or computed tomography) in combination with at least two of the following criteria: cough, sputum production, temperature > 38 °C or < 35 °C, hemoptysis, thoracic pain or auscultatory findings consistent with pneumonia. Pneumonia was classified as atypical by radiographic criteria and pulmonary IFD was diagnosed based upon the criteria reported by the European Organization for Research and Treatment of Cancer/Invasive Fungal Infections Cooperative Group and the National Institute of Allergy and Infectious Diseases Mycoses Study Group (EORTC/MSG) in (Pauw et al. 2008).

### Cytogenetic and molecular genetic analyses

Karyotype analyses by means of chromosome banding were performed with standard techniques, and karyotypes were described according to the International System for Human Cytogenetic Nomenclature (Brothman et al. 2005) Cytogenetic categorization into favorable, intermediate or adverse risk was performed on the basis of recommended criteria (International system for human cytogenomic nomenclature 2016).

The presence of *FLT3-ITD* and *NPM1* mutations was detected by PCR amplification of the corresponding region using genomic DNA followed by fragment analysis as previously described (Thiede et al. 2006).

Comprehensive molecular genetic analysis was performed in majority of AML patients undergoing intensive induction chemotherapy by next-generation sequencing (NGS) enabling a reliable prognostic stratification according to the European LeukemiaNet (ELN) 2017 classification (Stasik et al. 2021).

### Statistics

To test the statistical significance of differences in categorical data we applied the Chi-square test while for continuous variables the Student's *t* test was used. Differences between the Kaplan–Meier survival curves were evaluated by Log-rank (Mantel–Cox) test. *P* values of < 0.05 were considered as statistically significant. Statistical analyses were performed using GraphPad Prism 8.0.2 (GraphPad Inc.).

## Results

### Patients’ characteristics

This analysis comprises 220 consecutive AML patients including 118 Patients who received palliative AML treatment and 102 AML patients who underwent induction chemotherapy (Table 1). The median age (75 years; range 53–91 years) of AML patients receiving palliative treatment was significantly higher compared to AML patients with induction chemotherapy characterized by a median age of 61 years (range 25–76 years). The difference regarding age and the allocation to distinct treatment intensity associates with a significantly different distribution concerning the Eastern Collaborative Oncology Group (ECOG) performance status and the applied Charlson co-morbidity score between both patient subgroups. In detail, about half of AML patients (51.8%) undergoing palliative treatment could be attributed to ECOG 2 or higher compared to only 26.5% in the subgroup of AML patients receiving induction chemotherapy. Furthermore, the modified Charlson co-morbidity score (mCCI) directly correlated with the ECOG in both palliative AML patients (mCCI < 1 in 52.5% of patients versus mCCI > 2 in 47.5% of patients, respectively) and even more pronounced in those AML patients undergoing induction chemotherapy (mCCI < 1 in 78.4% of patients versus mCCI > 2 in 21.6% of patients, respectively).

There was no significant difference between both AML subgroups concerning AML history, previous therapy with hypomethylating agents (HMA) or distribution of AML subtypes according to the French-American-British (FAB) classification. Stratification according to the ELN 2017 classification revealed a significantly higher percentage of AML patients (25.5%) that could not be classified as favorable compared to the subgroup of AML patients receiving palliative treatment (25.5% versus 13.6%, *P* 0.025).

Blood counts did not demonstrate any significant difference between both AML subgroups in terms of WBC, Hb and platelet levels, bone marrow and peripheral blasts and especially with respect to absolute neutrophil counts (ANC) at AML primary diagnosis.

First-line treatment of both AML subgroups is summarized in the supplemental Table S1. In detail, the majority of palliative AML patients (82.2%) received HMA-based therapy either as single treatment or as combined treatment (e.g.; with the BCL-2 inhibitor venetoclax). Of patients receiving intensive AML therapy, 83 of 102 (81.4%) were treated with single induction chemotherapy according to the 7 + 3 schedule or the age-adapted OSHO protocol.

**Fig. 1:**
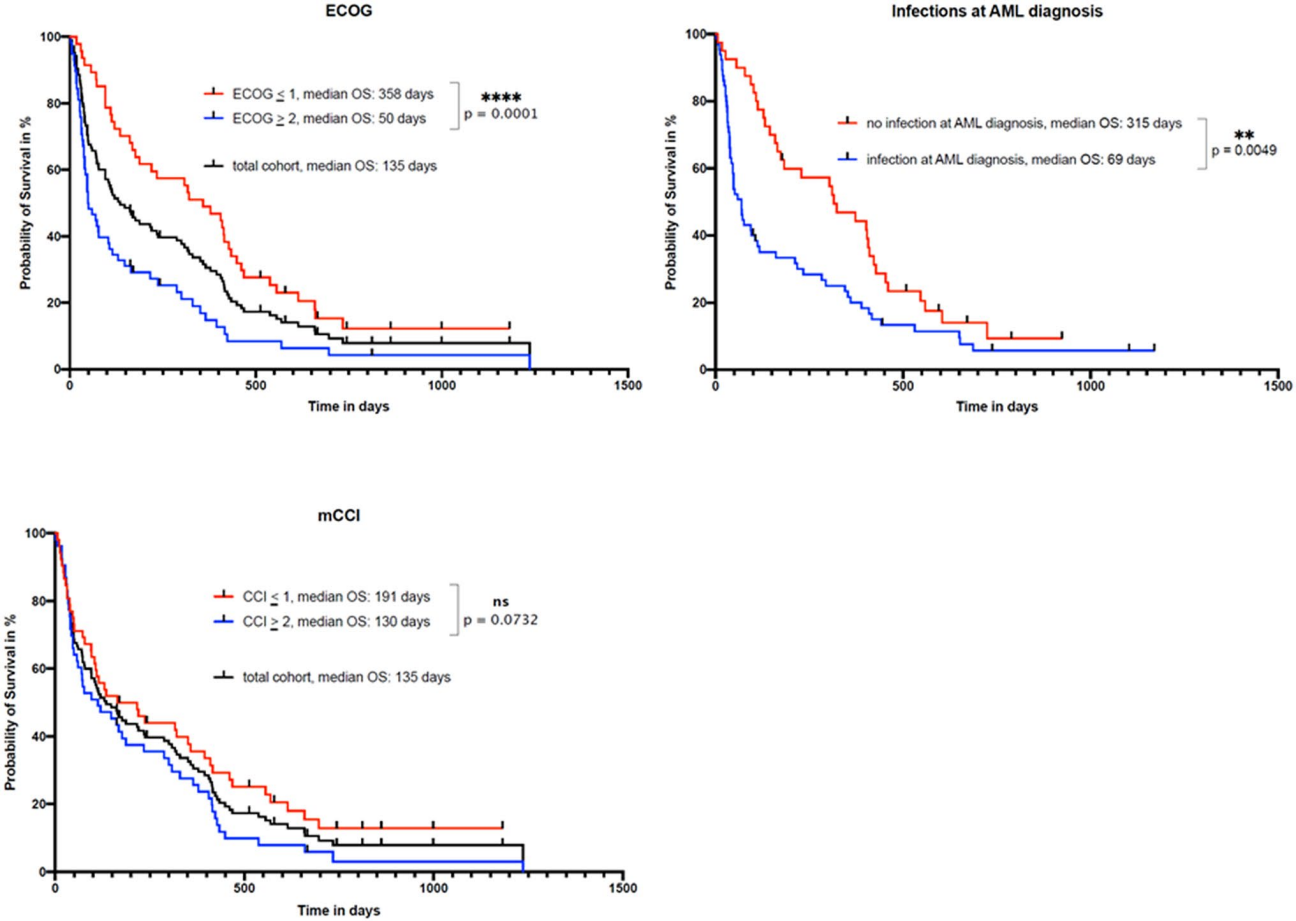
Kaplan–Meier estimates for overall survival (OS) dependent on the ECOG performance score **A** or on the modified Charlson comorbidity index **B** for AML patients undergoing palliative treatment

**Fig. 2:**
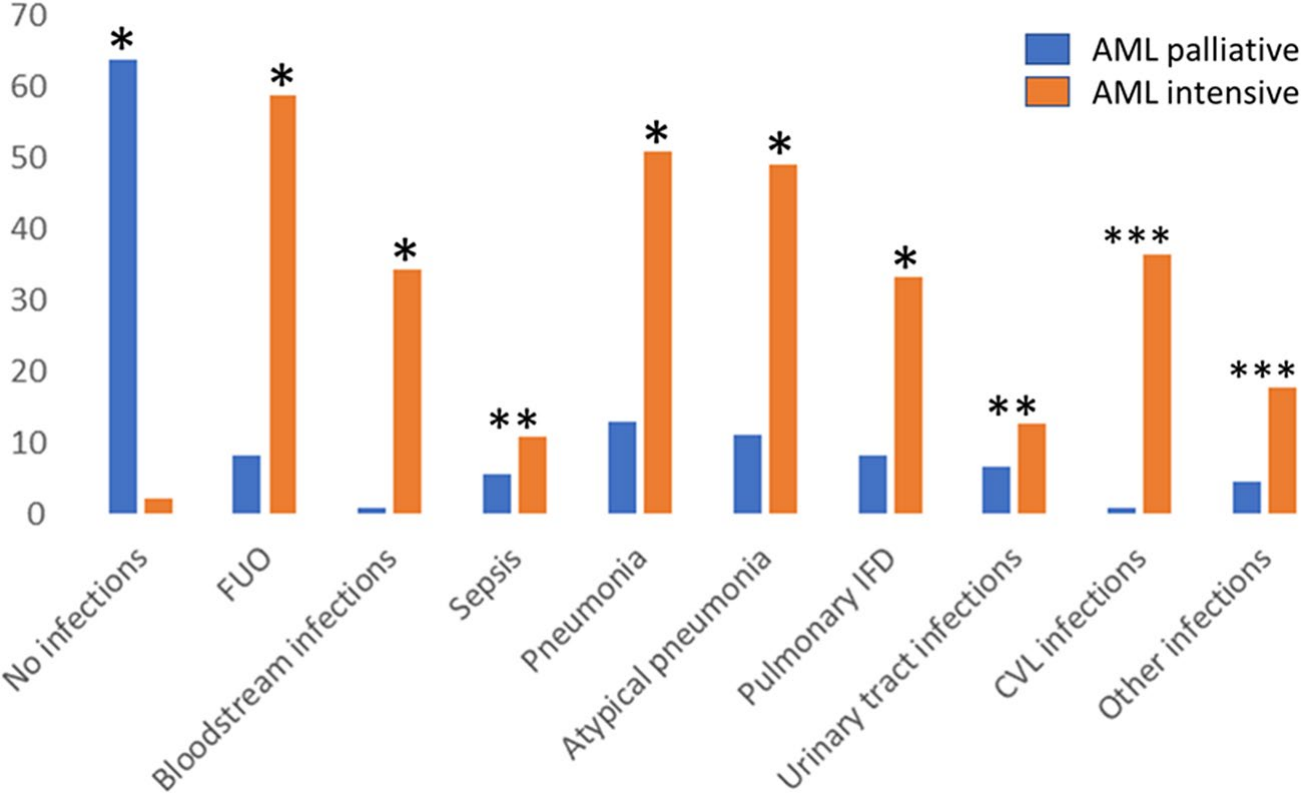
Comparison of infectious complications after initiation of palliative AML treatment (until day 100) or following AML induction chemotherapy. The percentage of distinct subsets of infections is indicated: * significant difference (*P* < 0.001), ** not significant, *** not applicable. A more detailed overview is given in Table S2

A detailed overview of AML patients receiving palliative treatment or undergoing induction chemotherapy, respectively, is provided in the CONSORT diagrams in the Supplement (Fig. 1S and 2S), respectively.

### Infectious complications at diagnosis of AML

A comprehensive analysis of all infections that were documented at the time of diagnosis and admission to the hospital was performed. Table 2 presents a classified overview of infectious complication at AML diagnosis for patients who were subsequently allocated to palliative treatment or induction chemotherapy of AML. Cumulative number of patients exceeds 118 patients in the palliative group and 102 patients who received induction chemotherapy, respectively, due to a possible overlap of infections or clinical situations (e.g., sepsis due to pneumonia).

**Table 2:** Spectrum of infectious complications at diagnosis of AML for both patient subgroups

	Palliative treatment	Induction chemotherapy	*P *value
	*N* = 118	*N* = 102	
Patients without infections, *n* (%)	47 (39.8)	64 (62.7)	< 0.001
FUO, *n* (%)	14 (11.9)	17 (16.7)	n.s
Bloodstream infection, *n* (%)	1 (0.8)	1 (1)	n.s
Sepsis, *n* (%)	5 (4.2)	2 (2)	n.s
Pneumonia	44 (37.3)	14 (13.7)	< 0.001
Typical Pneumonia	18 (15.3)	3 (2.9)	
Atypical pneumonia	26 (22.0)	11 (10.8)	
Atypical pneumonia	26 (22.0)	11 (10.8)	0.026
No IFD	12 (10.2)	5 (4.9)	
Possible IFD	13 (11.0)	6 (5.9)	
Probable IFD	1 (0.8)	0	
Other infections			
Urinary tract infection	6 (5.1)	–	0.021
Endocarditis	1 (0.8	–	
Bronchitis	1 (0.8)	–	
Oropharyngeal infection	–	3 (2.9)	
*Clostridium difficile* enteritis	1 (0.8)	–	
Soft tissue infection	1 (0.8)	3 (2.9)	

*FUO* Fever of unknown origin, *IFD* Invasive fungal disease

Patients allocated to palliative AML treatment presented a significantly higher probability of infections at diagnosis. In detail, 64 of 102 (62.7%) patients consecutively undergoing induction chemotherapy had no clinical signs of infections while only 47 of 118 (39.8%) of AML patients with palliative treatment presented without infections at diagnosis. In contrast, there was no difference concerning FUO, bloodstream infections and sepsis while more patients in the subgroup with subsequent palliative AML treatment had urinary tract infections (UTI) at diagnosis.

Detailed analysis of type of pneumonia especially considered the classification of potential invasive fungal disease (IFD) for all AML patients for this study. Significantly more patients attributed to palliative AML treatment did present with pneumonia at diagnosis of AML. Importantly, this observation does also translate in a significantly higher percentage of atypical pneumonia in these elderly AML patients. In detail, 37.3% of patients subsequently receiving palliative AML treatment presented with pneumonia including 22.0% of these patients with atypical pneumonia compared to 13.7% with pneumonia and 10.8% with atypical pneumonia, respectively, in the subgroup of AML patients allocated to induction chemotherapy.

Of note, the presence of infections at the time of AML diagnosis significantly correlates with the overall survival (OS) of palliative AML patients. In detail, the OS of elderly AML patients presenting without infectious complications at diagnosis was 315 days compared to only 69 days (*P* 0.0049) for patients with infections prior to start of palliative treatment in this intent-to-treat analysis (Fig. 1).

### Antifungal strategies in both AML subgroups

Table 3 summarizes both antifungal treatment in case of suspected IFD at diagnosis and different strategies of antifungal prophylaxis for both AML cohorts analyzed in this study. In detail, 12 of 14 patients subsequently allocated to palliative AML treatment presenting with pulmonary IFD could be evaluated for first-line antifungal therapy including 9 of these patients receiving secondary antifungal prophylaxis, while 5 palliative AML patients fulfilling the criteria of pulmonary IFD died either early at diagnosis (2 patients) or under treatment of pneumonia (3 patients).

**Table 3:** Antifungal strategies at diagnosis dependent on subsequent AML treatment intensity

	Palliative treatment	Induction chemotherapy
	*N* = 118	*N* = 102
Antifungal prophylaxis after AML diagnosis		
After IFD, *n* (%)	9 (7.6)	4 (3.9)
Posaconazole	2 (1.7)	4 (3.9)
Voriconazole	4 (3.4)	–
Isavuconazole	2 (1.7)	–
No prophylaxis	1 (0.8)	–
w/o IFD, *n* (%)	12 (10.2)	94 (92)
Posaconazole	11 (9.3)	93 (91)
Voriconazole	1 (0.8)	–
Isavuconazol	–	1 (1)
1st line antifungal treatment		
Liposomal amphotericin	6 (5.1)	2 (2)
Caspofungin	4 (3.4)	–
Posaconazole	2 (1.7)	–
Voriconazole	2 (1.7)	2 (2)

*AML* acute myeloid leukemia, *IFD* invasive fungal disease

With respect to antifungal treatment, liposomal amphotericin was applied in the majority of AML patients with fungal pneumonia in the palliative AML patient cohort and in two of four patients with pulmonary IFD prior to AML induction chemotherapy.

According to the presentation with either possible or probable IFD, patients with fungal infections at diagnosis were treated with secondary antifungal prophylaxis that regularly consisted of posaconazole or voriconazole (Table 3). Primary antifungal prophylaxis effectively targeting *Aspergillus spp.* were applied in only 12 additional patients subsequently undergoing palliative therapy, while the majority of patients within this AML subgroup received fluconazole prophylaxis in case of ANC below 1000 per µl. In contrast, almost all AML patients treated with induction chemotherapy received primary prophylaxis with posaconazole being effective against *Aspergillus spp.* Thus, there was a significant difference in terms of primary antifungal prophylaxis between AML patients receiving palliative treatment and those patients consecutively undergoing AML induction chemotherapy.

### Infectious complications after initiation of AML treatment

We next analyzed the spectrum of infectious complications after initiation of AML therapy to evaluate the impact of treatment intensity on the occurrence of severe infections in both AML treatment groups. The observation period was defined as follows: until day 100 after initiation of palliative AML treatment and until start of subsequent therapy (e.g. consolidation or allogeneic stem cell transplantation) following induction chemotherapy. 10 out of 118 AML patients of the palliative subgroup were lost of follow-up, so that the analysis of consecutive infectious complications included 108 patients of this AML subgroup. Figure 2 presents the comparison of infectious complications for AML patients either receiving palliative treatment or induction chemotherapy. The corresponding Table is included in the *Supplement* (Table S2).

Surprisingly, after successful treatment of infectious complications presenting at AML diagnosis the majority of patients receiving palliative AML treatment (63.9%) did not show further infectious complications until day 100 after initiation of AML therapy. This was significantly different compared to those AML patients with induction chemotherapy and does inversely correlate with the occurrence of both FUO and bloodstream infections. In detail, 60 out of 102 patients presented with at least one episode of FUO after initiation of induction chemotherapy and additional 35 patients had a bloodstream infection or fulfilled the criteria of sepsis (*n* = 11).

Importantly, the probability of bloodstream infections was significantly higher in the patient cohort undergoing induction chemotherapy as compared to those AML patients observed under palliative treatment. Positive blood samples were obtained in 40 patients resulting in 46 positive samples not considering two additional proven IFD each characterized by the detection of *Candida glabrata* blood stream infections. The spectrum of bacterial bloodstream infections for both AML treatment groups is shown in Figure S3. In detail, positive blood cultures detecting coagulase-negative Staphylococci spp. represent the major fraction in AML patients after induction chemotherapy while gut-associated bacteremia was due to both Gram-negative and Gram-positive pathogens. Importantly, *Enterococci spp.* isolated from blood culture specimens were exclusively tested positive for *E. faecium* in both AML treatment groups.

We next analyzed the impact of ANC on the frequency of clinically relevant infections in elderly patients. The potential impact of ANC at diagnosis on infectious complications in AML patients allocated to palliative treatment revealed no significant association for these patients at the time of AML diagnosis. Surprisingly, sepsis at diagnosis was only observed in those patients presenting with an ANC above 1000 per µl. Exploring the frequency of infectious complications for the time interval between AML diagnosis and day 100 for palliative AML patients, the probability of consecutive infections in general and for the occurrence of FUO was significantly higher in case of ANC below 1000 per µl (Supplement, Table S3).

**Fig. 3:**
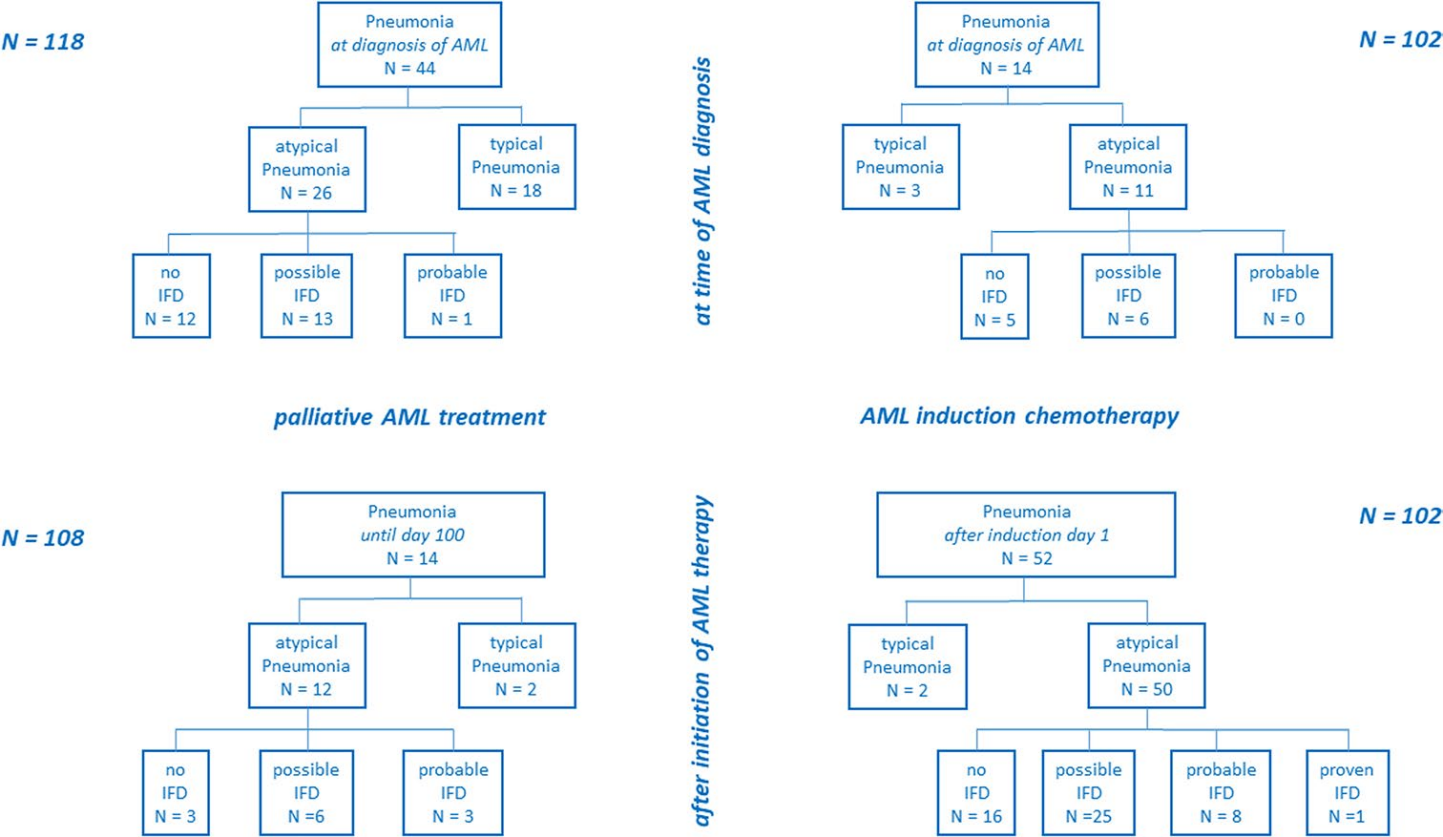
CONSORT diagram of time-dependent pneumonia subtypes for both patient groups. Distribution of absolute numbers of patients presenting with pneumonia either at the time of AML diagnosis (upper part) or after initiation of AML therapy (lower part) for patients allocated to palliative AML treatment (left) or induction chemotherapy (right)

The occurrence of pneumonia after initiation of AML therapy dependent on the applied treatment intensity was also studied. Figure 3 gives a detailed overview of distinct subsets of pneumonia documented either at diagnosis or after treatment initiation for both AML patient groups. A significantly higher rate of both pneumonia in general and atypical pneumonia in patients undergoing induction chemotherapy as compared to AML patients with palliative treatment is demonstrated: 51.0% versus 13.0% (*P* < 0.001) and 49.0% versus 11.1% (*P* < 0.001), respectively. Of note, the percentage of pulmonary IFD in the subgroup of atypical pneumonia is nearly identical between both patient subgroups at diagnosis—14 of 26 patients (53.8%) versus 6 of 11 patients (54.5%), respectively–and does also reveal a comparable distribution after treatment initiation: 9 of 12 patients (75.0%) versus 34 of 50 patients (68.0%), respectively. There is a clinically relevant and significantly higher risk of pulmonary IFD in AML patients undergoing induction chemotherapy. In detail, we could observe a total of 34 patients with pulmonary IFD including 1 proven IFD (*Rhizomucor spp.*) in this treatment group (34 of 102 patients, 33.3%), while only 9 Patients developed pulmonary IFD after initiation of palliative AML treatment (9 of 108 patients, 8.3%, *P* < 0.001).

The analysis of additional infections revealed no significant difference for such infectious complications as UTI or soft tissue infections. Because all patients undergoing AML induction chemotherapy had a CVL catheter in contrast to only few palliative AML patients, the comparison of CVL infections between both subgroups is not applicable. Importantly, there was no venous thrombosis in case of CVL infection. Of note, there were three non-pulmonary IFD including the already described bloodstream infections with *Candida glabrata* and one patient with sinusitis due to a suspected fungal infection.

An overview of most relevant infectious complications dependent on the time point of detection and on treatment intensity is presented in Figure S4 of the *Supplement*.

### ICU treatment and early mortality

We next analyzed the necessity of intensive care unit (ICU) treatment for both AML treatment groups. An overview of distinct ICU indications (e.g. vasopressor therapy or mechanical ventilation) for AML patients either receiving palliative therapy or undergoing induction chemotherapy is given in Table 4. In detail, 15 of 108 (13.9%) palliative AML patients were admitted to ICU mainly because of septic shock (*n* = 7) or severe pneumonia (*n* = 4). In AML patients treated with induction chemotherapy, indication for ICU treatment was documented in 18 of 102 (17.6%) patients including 7 patients with mechanical ventilation support due to severe pneumonia. Within the whole cohort of AML patients, 3 of 220 patients underwent leukapheresis. In 10 out of 15 (66.7%) palliative AML patients and 15 of 18 (83.3%) intensively treated AML patients, the acute clinical situation could be managed by ICU treatment enabling continuation of AML therapy.

**Table 4:** Intensive care unit (ICU) treatment indications and short-term outcome in AML patients of both subgroups

ICU indication	Palliative treatment *N* = 108	Induction chemotherapy *N* = 102
Sepsis	Death *n* = 1 Resolved *n* = 6	Death *n* = 1 Resolved *n* = 1
Pneumonia	Death *n* = 3 Resolved *n* = 1	Death *n* = 1 Resolved *n* = 6
Neurological/neurosurgical complications	resolved *n* = 2	Resolved *n* = 3
Pulmonary bleeding	Death *n* = 1	–
Leukapheresis	Resolved *n* = 1	Resolved *n* = 2
Toxicity/organ failure	–	Death *n* = 1 Resolved *n* = 2
Splenectomy	–	Resolved *n* = 1
Overall	Resolved *n* = 10 Death *n* = 5	Resolved *n* = 15 Death *n* = 3
Mortality	5 of 15 patients (33.3%)	3 of 18 patients (16.7%)

The early death rate defined as the mortality until day 60 after initiation of either palliative AML treatment or induction chemotherapy significantly differed between both subgroups (Table 5). In patients undergoing intensive AML treatment a low early mortality rate of 3.9% was demonstrated. In detail, two of four patients died of pneumonia, while septic shock or life-threatening toxicity resulted in lethal outcome in one patient each. A female patient with therapy-related AML following anthracycline-containing breast cancer chemotherapy died during ICU treatment of sepsis with predominant heart failure assumed because of anthracycline-induced cardiotoxicity.

**Table 5:** Mortality until day 60 after AML diagnosis for each treatment group

	Palliative treatment	Induction chemotherapy
	*N* = 108	*N* = 102
Mortality	34/108 (31.5%)	4/102 (3.9%)
Predominant progression of AML	8/34 (23.5%)	–
Pneumonia	15/34 (44.1%)	2 (50%)
No IFD	11/34 (32.4%)	–
IFD	1/34 (2.9%)	1 (50%)
With AML progression	3/34 (8.8%)	1 (50%)
Sepsis	8/34 (23.5%)	1 (25%)
w/o pneumonia	7/34 (20.6)	1 (100%)
With pneumonia	1/34 (2.9%)	
Bleeding	3/34 (8.8%)	–
Toxicity	–	1 (25%)

In contrast, 34 of 108 (31.5%) evaluable AML patients starting palliative therapy died between treatment initiation (e.g., HMA-based therapy) and day 60. As expected, there was a broad spectrum of causes of death in the palliative subgroup of AML patients with a potential overlap between clinical complications and AML progression in three patients and co-occurrence of sepsis and pneumonia in another AML patient. The main cause of death was pneumonia (44.1%) or sepsis (23.5%), respectively. Of note, we observed a very low rate of IFD with respect to early mortality in elderly AML patients (2.9% at day 60 and 4.5% at day 100, respectively).

### Impact of ECOG and co-morbidities on overall survival and infections

In elderly AML patients, a significant impact of the ECOG performance status at the time of AML diagnosis on overall survival (OS) was detected. In detail, median OS for patients with ECOG 0 or 1 was 353 days (range 17–1165 days) compared to 50 days (range 2–1219 days) in patients presenting with ECOG 2 or higher, respectively (Fig. 1, *P* < 0.001). In contrast, stratifying the cohort of elderly AML patients with respect to co-morbidities (mCCI < 1 vs. mCCI > 2) revealed only a trend in favor of patients with a low mCCI score with respect to the observed difference in OS: 165 days (range 6–1165 days) vs. 112 days (range 2–1219 days), respectively (*P* = 0.0732, Fig. 1).

Furthermore, there was a clear and significant association of an ECOG performance score of at least 2 and the occurrence of pneumonia including atypical pneumonia and especially for pulmonary IFD (Supplement, Table S4).

## Discussion

Treatment of AML has been improved by recent supportive care concepts and implementation of new targeted therapies of AML. However, AML therapy remains challenging in both younger and elderly AML patients. Treatment-related mortality (TRM) following induction chemotherapy of AML could be substantially reduced during the last 2 decades, e.g., by improvement of antifungal prophylaxis with posaconazole (Cornely et al. 2007; Othus et al. 2014).

Furthermore, treatment options beyond conventional “7 + 3” chemotherapy now consider mutation-specific or risk-adapted induction chemotherapy including midostaurin, gemtuzumab-ozogamicin or the liposomal application of cytarabine and daunorubicn (CPX-351) (Stone et al. 2017; Castaigne et al. 2012; Lancet et al. 2018) In patients who are not eligible for intensive treatment, epigenetic approaches with hypomethylating agents (azacytidine or decitabine) have been established (Dombret et al. 2015; Kantarjian et al. 2012) The addition of the BCL-2 inhibitor venetoclax can substantially improve the response rate and the overall survival of elderly AML patients as compared to single HMA treatment (DiNardo et al. 2020; Fleischmann et al. 2022).

We retrospectively analyzed a large cohort of consecutively treated AML patients at a tertiary hospital reflecting a series of AML patients mainly treated outside clinical trials. In this “intention-to-treat population”, we focused on the presentation and development of infectious complications dependent on different treatment intensity and distinct approaches of antifungal prophylaxis.

Elderly patients presented significantly more frequent with infectious complications at the time of AML diagnosis predominantly caused by pneumonia. Therefore, we addressed the question whether either absolute neutrophil counts, the ECOG performance status or the extension of comorbidities correlate with infectious complications. That significant correlation between higher ECOG score and the occurrence of pneumonia including pulmonary IFD is most likely explained by an interdependence of both clinical factors potentially contributing to a worse outcome in patient cohort (Caira et al. 2015) Our observation of a clinically relevant impact on overall survival with an inferior outcome of elderly AML patients presenting with infectious complications at diagnosis might explain the difference of median survival comparing results from clinical trials with such “real world” data as presented here.

Another potential factor for preexisting pneumonia at AML diagnosis might be attributed to an impaired function of innate immunity (e.g., in patients with antecedent MDS). Because there was no significant difference between our AML patient cohorts with respect to the percentage of de novo AML or hematological malignancies prior to AML diagnosis, respectively, we did not perform subgroup analysis focusing on this potential factor (Tey et al. 2021; Andréa et al. 2012).

In contrast, we could demonstrate a high rate of atypical pneumonia including a significantly higher proportion of pulmonary IFD in AML patients following induction chemotherapy. Several factors might contribute to the high incidence of pulmonary IFD in this well-defined cohort of AML patients: 1st, ANC are extremely low in AML patients undergoing induction chemotherapy; 2nd, the duration of severe neutropenia (ANC < 500 per µl); 3rd, the treatment-associated toxicity of conventional chemotherapy.

In detail, despite there was no significant difference concerning ANC at diagnosis between both AML patient groups, subsequent reduction of neutrophil counts can be expected to be much more pronounced after application of induction chemotherapy as compared to palliative AML treatment (e.g. with hypomethylating agents). Furthermore, the duration of usually undetectable neutrophils following induction chemotherapy depends on such important factors as age, antecedent hematological malignancy and especially on the response to the first induction cycle (Caira et al. 2015; Pagano et al. 2010; Hahn-Ast et al. 2010) .

In addition, the impact of treatment-related toxicity represents another factor that might contribute to a higher rate of pneumonia. Especially considering the presence of opportunistic pathogens (e.g. *Aspergillus spp*. or *Pneumocystis jirovecii*) at low levels in the bronchoalveolar system in combination with chemotherapy-induced pulmonary tissue damage might contribute to the high rate of atypical pneumonia (Traber et al. 2017; Chang et al. 2018).

The especially high number of patients attributed to possible IFD according to the EORTC/MSG guidelines is also due to the strict diagnostic work-up in our AML population. In detail, all patients with neutropenic fever received at least one chest computed tomography scan with a high percentage of subsequent bronchoalveolar lavage (BAL) in case of pulmonary infiltrates. Both diagnostic strategies led to a high detection rate especially of possible IFD being classified by characteristic infiltrates or a combination with distinct clinical symptoms, while the cumulative incidence of proven and probable IFD was well comparable with recently published studies (Wang et al. 2019; Wasylyshyn et al. 2021; Gomes et al. 2014).

Of note, significantly less patients presented with infections after initiation of palliative AML treatment compared to the cohort receiving induction chemotherapy. Both the occurrence of febrile neutropenia or bacteremia and the observation of a significantly higher incidence of pneumonia including pulmonary IFD following induction chemotherapy could also be demonstrated by Jalbut and colleagues analyzing a cohort of 172 AML patients (Jalbut et al. 2018) To our best knowledge, this study represents one of the most comprehensive comparisons of infectious complication in AML patients either receiving palliative treatment or induction chemotherapy.

In a recently published study investigating fungal infections in AML patients treated with HMA and venetoclax, there was a low risk of fungal infections despite distinct antifungal strategies (Aldoss et al. 2019) The population of elderly AML patients included in the retrospective analysis presented by Aldoss and colleagues is comparable with our patient population undergoing palliative AML treatment. In detail, the overall rate of 8.4% patients with pulmonary IFD was quite similar to the observation of either proven or possible IFD in the study published by Aldoss et al. indicating 12.6% patients with IFD.

The application of *Aspergillus spp*.-directed antifungal strategies in patients undergoing palliative AML treatment should be considered in patients either presenting with low neutrophil counts at diagnosis or in case of treatment-associated prolonged severe neutropenia.

Analysis of pathogens in positive blood stream specimens revealed a high percentage of coagulase-negative *Staphylococci spp.* in those patients undergoing AML induction chemotherapy correlating with the application and potential infection of central venous catheters. Considering coagulase-negative *Staphylococci spp.*, there was a higher rate of blood stream infections compared to a large cohort of AML patients published by Conn and colleagues (Conn et al. 2017) The detection of *Enterococci spp.* in blood stream samples was restricted to *E. faecium* being of clinical relevance with respect to the empiric antibiotic strategies in neutropenic patients. The observation that Gram-positive pathogens were most frequently detected in culture-positive blood samples has also been shown in a large series of AML and MDS patients treated with decitabine previously published by Ali and co-workers (Ali et al. 2017).

We did also analyze the spectrum of indications and the outcome after ICU treatment in both AML patient subgroups. Even in patients receiving palliative AML treatment, ICU treatment had a favorable short-term outcome in two-thirds of patients necessitating ICU treatment. This provides a clear rationale to include ICU regimens in certain clinical situations despite a palliative treatment approach. Especially time-limited vasopressor therapy should be considered in case of sepsis in elderly AML patients enabling either bridging to initiation or continuation of AML therapy in these patients. This is in line with previous reports analyzing the impact of ICU treatment approaches in AML patients (Hammond et al. 2021; Mottal et al. 2020).

The decision which treatment intensity should be applied to an elderly AML patient should be made based on individual factors including age, comorbidity, performance status, etiological and genetic features of AML. Furthermore, a higher rate of early death following induction chemotherapy because of treatment-related complications (e.g., severe infections) has been observed (Krug et al. 2010) Of note, retrospective comparison of induction chemotherapy versus HMA-based treatment in elderly AML patients demonstrated a similar outcome due to a higher early death rate following induction chemotherapy (Quintás-Cardama et al. 2012).

We are aware of the limitations of our study due to its retrospective analysis and a heterogeneity especially of the cohort including AML patients undergoing palliative treatment. Thus, the treatment-associated toxicity is not balanced between distinct subgroups of palliative AML patients. Furthermore, the percentage of subsequent BAL diagnostics in case of suspected pulmonary infiltrates was higher in patients undergoing induction chemotherapy compared to patients receiving palliative AML treatment (e.g. due to co-morbidities). Despite the low rate of possible IFD diagnosed in the cohort of elderly AML patients, this represents another potential limitation of our retrospective analysis.

Taken together, pneumonia including atypical pneumonia can be significantly more often detected at diagnosis in elderly AML patients subsequently allocated to palliative treatment. In contrast, after initiation of AML therapy the rate of infectious complications and especially bloodstream infections, fever of unknown origin or fungal infections is significantly higher following induction chemotherapy. Importantly, in elderly AML patients the ECOG performance status and the presence of infections at diagnosis represent clinically relevant prognostic factors concerning the outcome of patients undergoing palliative treatment.

## Supplementary Information

Below is the link to the electronic supplementary material.

**Figure S1: CONSORT diagram of AML patients (n=118) with palliative treatment.** Illustration of antecedent hematological disease or specific therapy prior to diagnosis of AML and distribution of first-line AML therapy in patients allocated to palliative AML treatment. (PPTX 44 kb)

**Figure S2: CONSORT diagram of AML patients (n=102) with induction chemotherapy. **Overview of antecedent hematological disease or specific therapy prior to diagnosis of AML, distribution of hematological response and subsequent treatment of patients undergoing induction chemotherapy as first-line treatment of AML. (PPTX 195 kb)

**Figure S3: Spectrum of pathogens obtained from blood culture samples dependent on AML treatment intensity. **Distribution of positive blood culture specimens indicating the spectrum of isolated bacteria in febrile patients after palliative AML treatment (A) or following induction chemotherapy (B). (PPTX 44 kb)

**Figure S4: Comparison of most relevant infectious complications at diagnosis and after initiation of AML treatment for both patient subgroups.** Percentage of most relevant infectious complications at diagnosis of AML or after initiation of AML therapy for patients receiving palliative AML treatment or induction chemotherapy. (PPTX 54 kb)

Supplementary file5 (DOCX 16 kb)
