# Supplementary figures and images for: Impact of treatment intensity on infectious complications in patients with acute myeloid leukemia
Source: J Cancer Res Clin Oncol. 2022 May 18;149(4):1569–83. doi: 10.1007/s00432-022-03995-2 (PMC10020242; doi:10.1007/s00432-022-03995-2)

## Slide 1
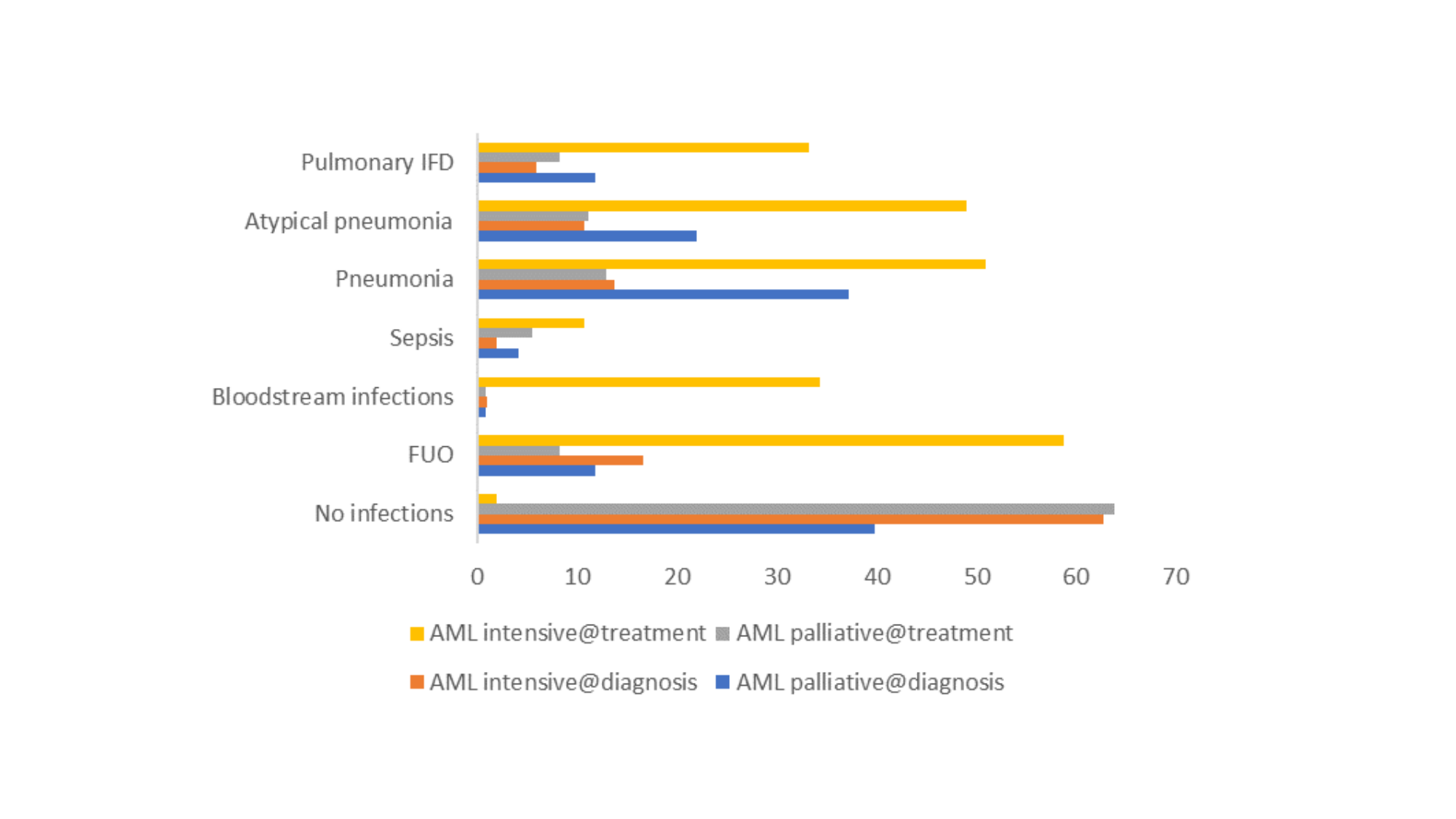

#

Supplement: Supplementary file 1 — Figure S1: CONSORT diagram of AML patients (n=118) with palliative treatment. Illustration of antecedent hematological disease or specific therapy prior to diagnosis of AML and distribution of first-line AML therapy in patients allocated to palliative AML treatment. (PPTX 44 kb) [file 432_2022_3995_MOESM1_ESM.pptx]

## Slide 1
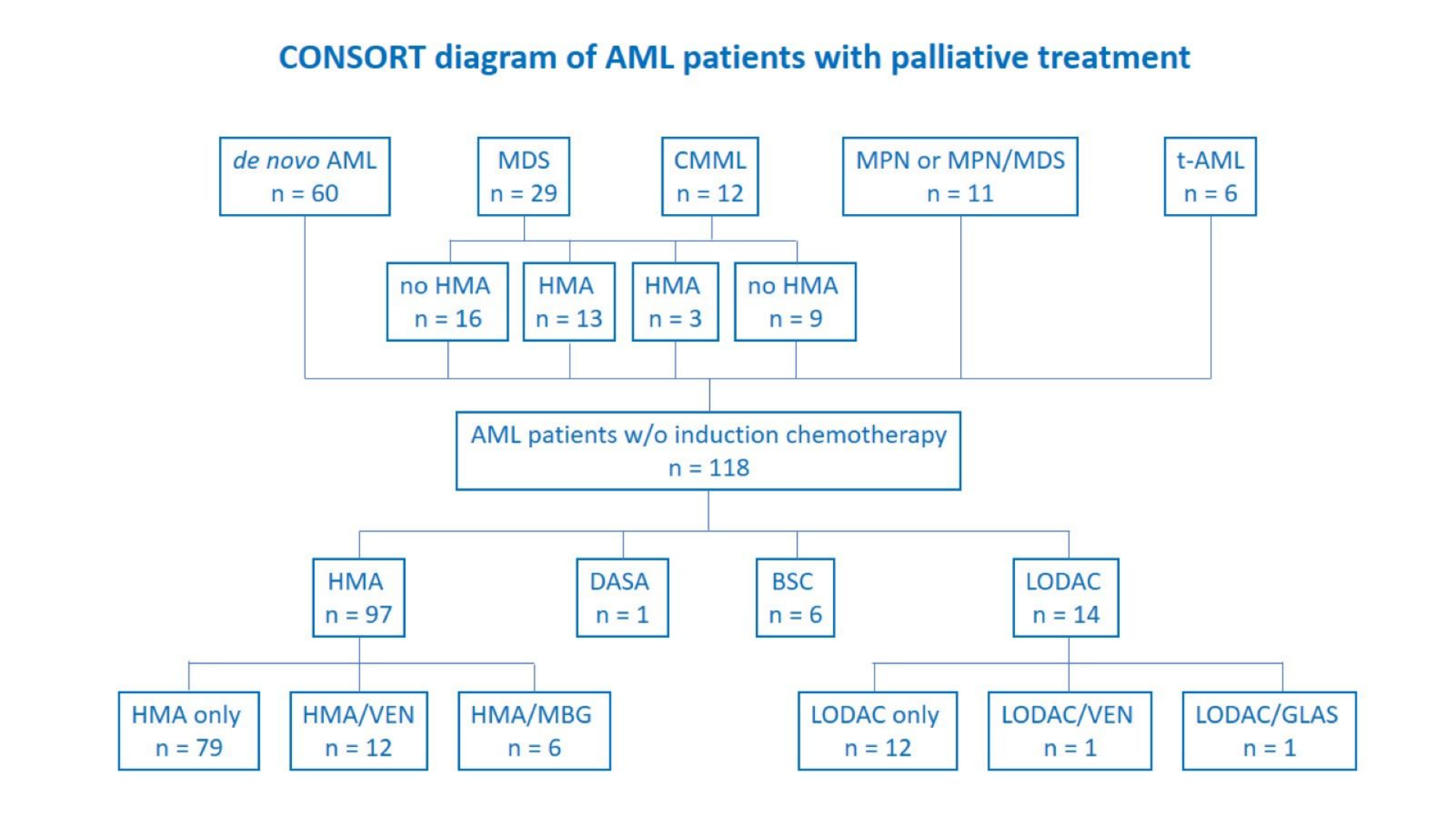

#

Supplement: Supplementary file 2 — Figure S2: CONSORT diagram of AML patients (n=102) with induction chemotherapy. Overview of antecedent hematological disease or specific therapy prior to diagnosis of AML, distribution of hematological response and subsequent treatment of patients undergoing induction chemotherapy as first-line treatment of AML. (PPTX 195 kb) [file 432_2022_3995_MOESM2_ESM.pptx]

## Slide 1
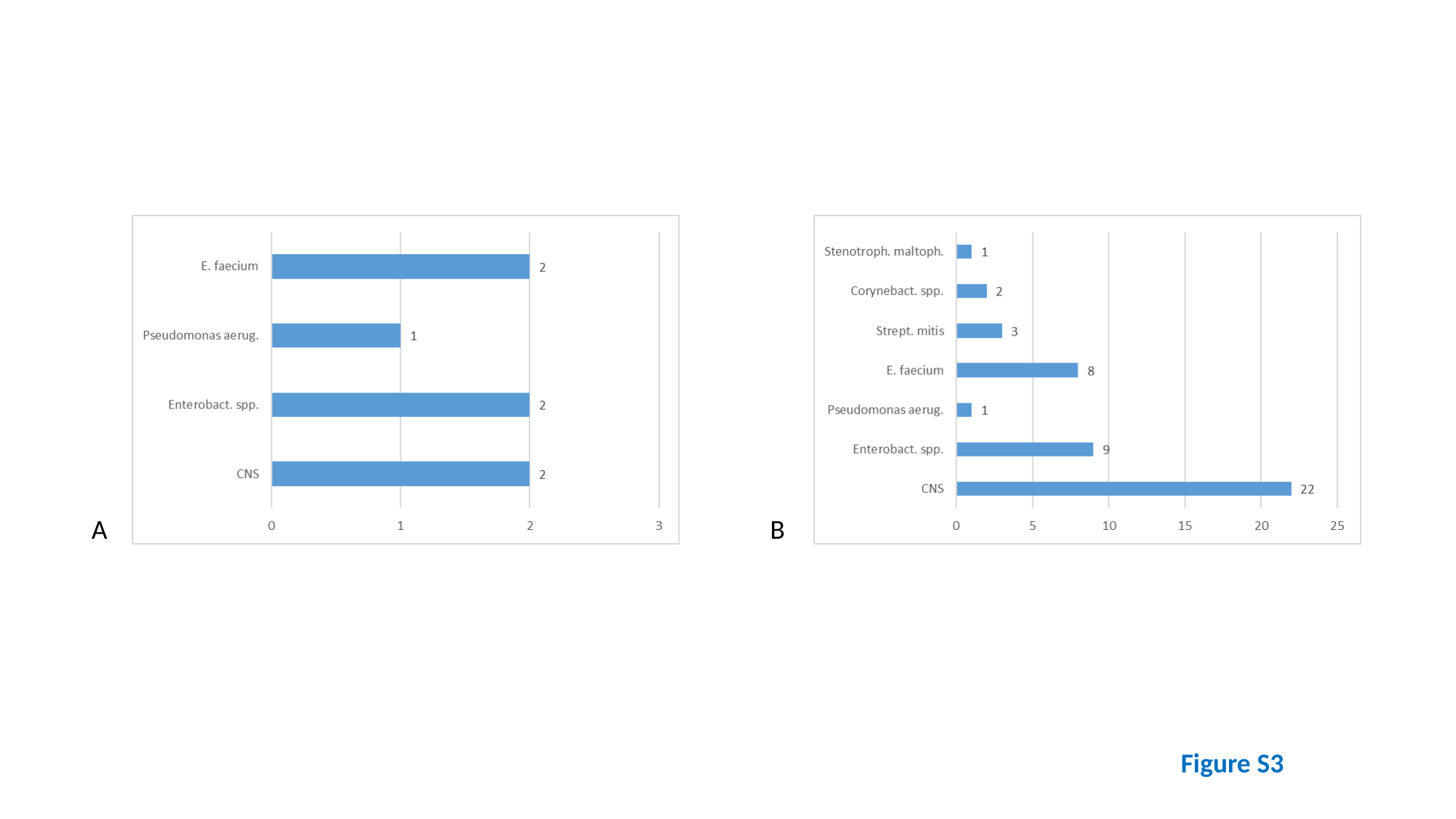

### Chart
| Category |
|---|
A
B
Figure S3

Supplement: Supplementary file 4 — Figure S4: Comparison of most relevant infectious complications at diagnosis and after initiation of AML treatment for both patient subgroups. Percentage of most relevant infectious complications at diagnosis of AML or after initiation of AML therapy for patients receiving palliative AML treatment or induction chemotherapy. (PPTX 54 kb) [file 432_2022_3995_MOESM4_ESM.pptx]
