# Supplementary material for: Impact of treatment intensity on infectious complications in patients with acute myeloid leukemia
Source: J Cancer Res Clin Oncol. 2022 May 18;149(4):1569–83. doi: 10.1007/s00432-022-03995-2 (PMC10020242; doi:10.1007/s00432-022-03995-2)
Supplement: Supplementary file 3 — Figure S3: Spectrum of pathogens obtained from blood culture samples dependent on AML treatment intensity. Distribution of positive blood culture specimens indicating the spectrum of isolated bacteria in febrile patients after palliative AML treatment (A) or following induction chemotherapy (B). (PPTX 44 kb) [file 432_2022_3995_MOESM3_ESM.pptx]

## Slide 1
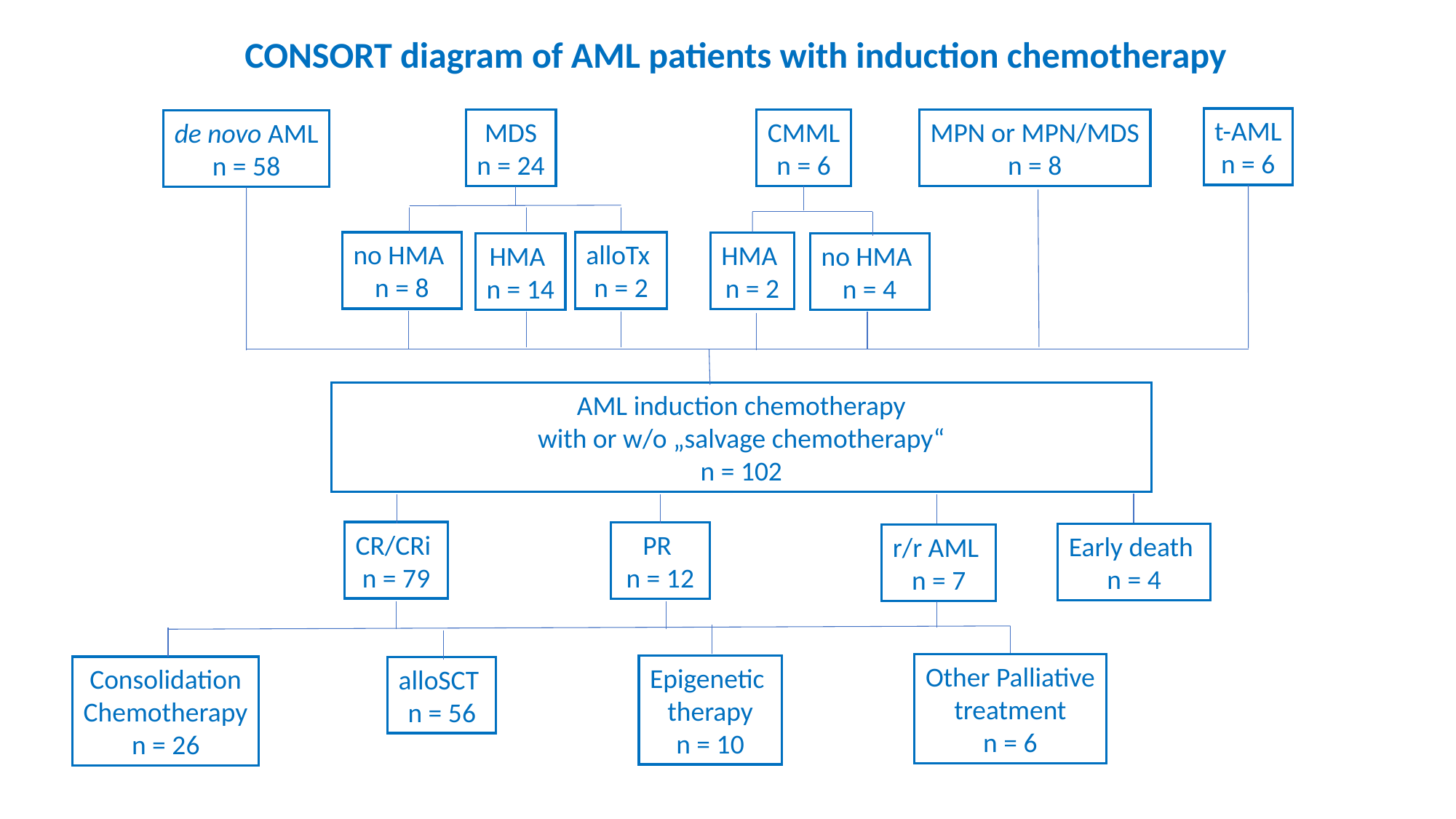

CONSORT diagram of AML patients with induction chemotherapy
t-AML
n = 6
CMML
n = 6
MDS
n = 24
MPN or MPN/MDS
n = 8
de novo AML
n = 58
no HMA
n = 8
alloTx
n = 2
HMA
n = 2
no HMA
n = 4
HMA
n = 14
AML induction chemotherapy
with or w/o „salvage chemotherapy“
n = 102
CR/CRi
n = 79
PR
n = 12
Early death
n = 4
r/r AML
n = 7
Other Palliative
treatment
n = 6
Epigenetic
therapy
n = 10
Consolidation
Chemotherapy
n = 26
alloSCT
n = 56
