# Supplementary material for: Impact of treatment intensity on infectious complications in patients with acute myeloid leukemia
Source: J Cancer Res Clin Oncol. 2022 May 18;149(4):1569–83. doi: 10.1007/s00432-022-03995-2 (PMC10020242; doi:10.1007/s00432-022-03995-2)
Supplement: Supplementary file 5 — Supplementary file5 (DOCX 16 kb) [file 432_2022_3995_MOESM5_ESM.docx]

**SUPPLEMENT**

|  | Palliative Treatment  N = 118 | Induction chemotherapy  N = 102 |
| --- | --- | --- |
| Cytoreductive chemotherapy, n (%)  Hydroxyurea  Cytarabine | 43 (36.4)  26 (22.0)  17 (14.4) | 48 (47.1)  23 (22.6)  25 (24.5) |
| 1^st^ line treatment of AML, n (%)  HMA-based  LODAC-based  Dasatinib  BSC only | 97 (82.2)  14 (11.9)  1 (0.8)  6 (5.1) |  |
| 1^st^ line treatment of AML, n (%)  single 7+3 induction  double 7+3 induction  OSHO < 60 years  OSHO > 60 years  CPX-351  Salvage induction with MitoFLAG |  | 24 (23.5)  18 (17.7)  15 (14.7)  44 (43.1)  1 (1)  11 (10.8) |

**Table S1: Overview of first line AML therapy for both patient subgroups.** Abbreviations: BSC, best supportive care; CPX-351, liposomal formulation of induction chemotherapy (Vyxeos^R^); HMA, hypomethylating agent; LODAC, low-dosed cytarabine; OSHO, East German Study Group of Hematology and Oncology

|  | AML palliative  until day 100  N = 108 | AML intensive  after induction  N = 102 | *P*  value |
| --- | --- | --- | --- |
| Patients without infections, n (%) | 69 (63.9) | 2 (2.0) | <0.001 |
| FUO, n (%) | 9 (8.3) | 60 (58.8) | <0.001 |
| Bloodstream infection, n (%) | 1 (0.9) | 35 (34.3) | <0.001 |
| Sepsis, n (%) | 6 (5.6) | 11 (10.8) | n.s. |
| Pneumonia, n (%)  typical Pneumonia  atypical pneumonia | 14 (13.0)  2 (1.9)  12 (11.1) | 52 (51.0)  2 (2.0)  50 (49.0) | <0.001 |
| Atypical pneumonia, n (%)  no IFD  Possible IFD  Probable IFD  Proven IFD | 12 (11.1)  3 (2.8)  6 (5.6)  3 (2.8)  0 | 50 (49.0)  16 (15.7)  25 (24.5)  8 (7.8)  1 (1.0) | <0.001  <0.001 |
| Other infections, n (%)  Urinary tract infection  Oropharyngeal infections  Mediastinitis  Bronchitis  *Cl. difficile* enteritis  Soft tissue infection  CVL infections  Non-pulmonary IFDs  Possible IFD  Proven IFD | 7 (6.5)  0  0  1 (0.9)  1 (0.9)  2 (1.9)  1 (0.9)  1 (0.9)  - | 13 (12.7)  7 (6.9)  1 (1.0)  0  2 (2.0)  6 (5.9)  37 (36.3)  -  2 (2.0) | n.s.  n.a. |

**Table S2: Infectious complications following palliative AML treatment (until day 100) or AML induction chemotherapy.** Abbreviations: CVL, central venous line; FUO, fever of unknown origin; IFD, invasive fungal disease; n.s., not significant; n.a. not applicable

|  | at | diagnosis |  | until | day 100 |  |
| --- | --- | --- | --- | --- | --- | --- |
| N = 108 | ANC < 1000  per µL  N = 43 | ANC > 1000  per µL  N = 65 | *P* | ANC < 1000  per µL  N = 43 | ANC > 1000  per µL  N = 65 | *P* |
| Patients without infections, n (%) | 18 (41.9) | 23 (35.4) | n.s. | 19 (44.2) | 50 (77.0) | <0.001 |
| FUO, n (%) | 5 (11.6) | 6 (9.2) | n.s. | 6 (14.0) | 1 (1.5) | 0.01 |
| Bloodstream  infection, n (%) | 1 (2.3) | 0 | n.s. | 1 (2.3) | 0 | n.s. |
| Sepsis, n (%) | 0 | 5 (7.7) | 0.062 | 4 (9.3) | 2 (3.1) | n.s. |
| Pneumonia, n (%)  typical Pneumonia  atypical pneumonia | 13 (30.2)  5 (11.6)  9 (20.9) | 27 (41.5)  13 (20.0)  14 (21.5) | n.s. | 6 (14.0)  4 (9.3)  2 (4.7) | 5 (7.7)  2 (3.1)  3 (4.6) | n.s. |
| Atypical pneumonia, n (%)  no IFD  Possible IFD  Probable IFD | 9 (20.9)  3 (7.0)  5 (11.6)  1 (2.3) | 14 (21.5)  8 (12.3)  6 (9.2)  0 | n.s. | 2 (4.7)  0  1 (2.3)  1 (2.3) | 2 (3.1)  0  2 (3.1)  1 (1.5) | n.s. |

**Table S3: Occurrence of Infectious complications dependent on ANC until day 100 of palliative AML therapy**

| N = 108 | ECOG < 1  N = 50 | ECOG > 2  N = 58 | *P* |
| --- | --- | --- | --- |
| Patients without infections, n (%) | 31 (62.0) | 10 (17.2) | <0.001 |
| FUO, n (%) | 7 (14.0) | 4 (6.9) | n.s. |
| Bloodstream infection, n (%) | 1 (2.0) | 0 | n.s. |
| Sepsis, n (%) | 1 (2.0) | 4 (6.9) | n.s. |
| Pneumonia, n (%)  typical Pneumonia  atypical pneumonia | 7 (14.0)  5 (10.0)  2 (4.0) | 33 (56.9)  13 (22.4)  21 (36.2) | <0.001 |
| Atypical pneumonia, n (%)  no IFD  Possible IFD  Probable IFD | 2 (4.0)  2 (4.0)  0  0 | 21 (36.2)  9 (15.5)  11 (19.0)  1 (1.7) | <0.001 |

**Table S4: Occurrence of Infectious complications dependent on ECOG at diagnosis**
